# Irregularity of the Teeth and Treatment

**Published:** 1875-09

**Authors:** N. E. Beach


### IRREGULARITY OF THE TEETH AND TREATMENT.

BY N. E. BEACH, D. D. S.

*Read before the Tenn. State Dental Society.*

In the selection and arrangement of subjects for discussion, at this, the ninth annual meeting of this association, I am left to select my own; and, at the same time, every department of practical interest to the profession has been well provided for, except that of the treatment of irregularities of the teeth, with its causes, etc.

It does not seem to me that this is a subject which is pressing itself upon the dentist, with such alarming frequency as to demand their most serious consideration.

I do not believe there is in any branch of practice, a greater necessity for scientific investigation than in this.

However much we may shrink from undertaking to cor rect the irregular development of teeth, the fact still exists, that is, the necessity for such labor, and skill, is on the increase.

Is it true, that so much of the timidity of dentists in the treatment of irregularities of the teeth, is owing to a lack of the inventive skill required to make successful appliances? Or is it the tediousness of the operation, together with the uncertain prognosis? or is it because of the absence of appreciation by the public of this, the highest order of professional skill, together with an unwillingness to compensate the dentist for the time, labor, mental and physicial, expense, etc., incurred in the successful treatment of such cases?

If the former, then in my opinion, it would be wise in the dentist to properly instruct such of his patients as may require treatment, and recommend to them, such a one, as in their judgment may be both skillful and willing enough to undertake such labor.

I do not consider it a discredit to any professional man not to be able to do everything pertaining to his profession with equal facility, but, on the contrary, it is manly and wise to say to your patient, that such an operation can be more skillfully done by some one else, and if necessary, go with them and state the object of your visit in such a way as to inspire confidence in all concerned.

How many dentists are there who never extract a tooth or make an artifical denture; yet they stand high in the scientific world. How many M. D's. who never perform a surgical operation, except of minor importance? How many legal gentleman who never write a brief, because of inability to write a legible hand. Yet such men stand high in their professions.

I feel thankful that the highest order of scientific investigation is now being directed more to the prevention of disease than its remedy, and in order to successfully combat the increasing tendency it is of paramount importance, to first know the causes which produce the irregular development of the human teeth. The researches of Dr N. W. Kingsley of New York, have given us a paper on this subject, published in Dental Cosmos. Vol. xvii. No. 4, which does credit to himself, and the profession of which he is a worthy member, but inasmuch as my object in presenting this paper is to deal with known facts, and offer some suggestions in the treatment of cases belonging to special classes of irregularity, I will make no comment on Dr Kingsley's paper.

We have but two general classes of irregularity of teeth, one inherited, the other accidental.

By inherited irregularity I mean all similarity of arrangement of the teeth of the child to that of either parent. It is a fact well established by obsərvation, that the transmission from parent to offspring of pecular characteristics, either normal or abnormal through successive generations is common. The same movements of the body, tones of voice, color of hair and eyes, or general expression in features, and also in disease are things of almost daily observation.

Well has it been said by Prof. McQuillen that, "the tendency to the reproduction of individual peculiarities in the descendants of human beings, is in no part of the organism made more markedly manifest than in the structure, size, form, and relative position of the teeth." We see that, with wonderful exactness the teeth of children, and even grand-children, are in shape and position like that of the parents. Aside from this similarity of form and arrangement, there is another inherent irregularity caused by the child inheriting the maxillary bones from one parent, and teeth from the other. This is a theory which has met with much favor, and seems logically true, but in reality there is some doubt whether nature would so far depart from her usual course, as to develop the maxillary bones in size and form, like one parent, and their appendages, the teeth and alveolar ridge, like that of the other. To do this, would certainly require an unnatural divsion of the circulating medium by which these parts are formed. Admitting this to be true in fact, then it must follow that the irregularity will be by the teeth being of the larger type, while the maxillæ arc the smaller.

Such a state of things, would produce either too much prominence of the anterior teeth, or crowding out of the arch one or both of the canine teeth. If the former be the case, then in order to remedy the defect, room should first be made by extracting on each side, such a tooth as the condition they are in would indicate, and applying mechanical pressure in the manner described in a series of articles published by Dr. Kingsley in "Johnston's Dental Miscellany" for 1874.

It is sometimes necessary in order to prevent the teeth from slipping by each other, when the pressure is put on the incisors alone, to resort to other means than those mentioned by Dr. K. That which I have found most successful is, to make a plate to envelope all the teeth in the upper jaw except those to be acted on, and on this plate, fasten hooks, or cut slots on both sides of the alveolar ridge as far back as is necessary to get the requisite tension on the rubber tubing. Then cut pieces of tubing of such size as is believed to be of proper strength, pass it over the hooks or slots and bring it foıward and pass it over the tooth to be moved, by this means they may be carried inward and backward, one on each side at a time until they are all in position.

There is another and in some cases, at least a better way than the plan described by Dr. K. That is to take a small band of gold long enough to cover the four anterior teeth, with a small hole in each end, through each of these holes pass a piece of waxed floss silk and tie it in a piece of rubber tubing. On each side of the buccal portion of such a plate, as I have before described, fasten a flattened hook, pass the tubing already tied to the ends of the band, over these hooks, insert the plate in the mouth, and draw the metal band forward place it in position on the outside of the anterior teeth.

This appliance is more easily managed by the patient, acts with more uniform pressure and is less in the way of the tongue than the other.

If the band is inclined to slip too near the gum, fasten it in position with a ligature of floss silk, or silver wire.

If the teeth are to much crowded, with no unnatural protrusion, it will only be necessary to remove one tooth on each side so as to give plenty of room. If this is done in time, they usually regulate themselves.

The next deviation from correct articulation, is the reverse of the one above desci ibed, that is, where one or more of the superior incisors fall inside of the inferior ones. It was my purpose to speak of the causes most likely to produce the different forms of irregularity, but it will make my paper too long, therefore I will only speak of such forms as are most frequently met with, and the treatment. This is quite frequent and easily remedied. The best means is not to rely on the old method as taught in most of the books, viz; putting an inclined plane on the lower teeth, but to make a rubber plate, so as to cover most of the roof of the mouth, and the posterior teeth, with a rim passing around the labial portion of the anterior teeth. This should be done so as to give plenty of room to move such teeth as are to be acted on outward, as far as necessary without bringing them in too close contact with said rim; then by cutting holes, or slots in the rim, and applying rubber ligature, either of French tubing, or strips of rubber dam, the teeth may be drawn out in position in a few days. It will only be necessary to hold them in their new position eight to ten days, when it will be found that the lower teeth will keep them there.

Another form, which requires frequent attention is, cases in which the bicuspids come through on the palatine side of the deciduous molars, causing a narrowing of the arch. When this is the case with these teeth on both sides of the arch alike, an easy and simple method of forcing them in position, is to swage a metal plate so as to fit closely against the teeth to be moved, and spring it into position, then, by straightning the curvature of the plate every day; and again springing it in position they may generally be easily spread, so as to take their normal position. The change in the shape of the plate will be found to vary, nearly in accordance with the change in the shape of the arch.

When the abnormal condition exists on one side alone, the plate should be made of rubber, so as to fit closely all the teeth on the normal side, and the deviating teeth acted on by means of a suitable jackscrew, so arranged as to use all the teeth on the normal side as a base from which to make pressure on the deviating tooth or teeth. In all cases, the judgment of the operator should designate the length of time it is necessary to wear a retaining plate, when it is necessary at all.

The next class, of which I will speak, is the kind I term accidental irregularity. By the term accidental I include all deviations from a natural arrangement, when there is no indication to lead to the belief that it is in any way hereditary.

When the parents and grandparents, as far as known, all have symmetrically proportioned teeth, evenly arranged, any departure from such an arrangement in their posterity is unnatural and must, of necessity, be accidental.

In this class we find the most frequent departure from a normal condition, to be a twisted condition of the incisor teeth most frequently, but one or two, but occasionally all four of the superior incisors, while the inferior ones are perfectly normal.

The treatment for turning these crooked teeth, as they are commonly called, has in my opinion been much abused by some, who recommend what they designate as "torsion."

The description given of this method, shows that the operation is performed in a few seconds, with the aid of a pair of forceps. While this may prove to be successful in some instances, I regard it as unsafe, and likely to result in the destruction of the dental pulp, and greatly lessen the durability and usefulness of the tooth. A much safer and better method, is by the use of metallic rings with small hooks on them and rubber ligatures, used in the manner described in a paper, I read before this body at our last meeting, when I fully demonstrated by models and appliances, exhibited on that occasion.

In the foregoing remarks, I have not sought to introduce anything new or startling, but simply to give you a brief paper, showing what in my opinion and practice has proven most successful, simple and useful in the treatment of such cases of irregularity, as are of most frequent occurrence.

